# 3D black blood VISTA vessel wall cardiovascular magnetic resonance of the thoracic aorta wall in young, healthy adults: reproducibility and implications for efficacy trial sample sizes: a cross-sectional study

**DOI:** 10.1186/s12968-016-0237-2

**Published:** 2016-04-14

**Authors:** Anouk L. M. Eikendal, Björn A. Blomberg, Cees Haaring, Tobias Saam, Rob J. van der Geest, Fredy Visser, Michiel L. Bots, Hester M. den Ruijter, Imo E. Hoefer, Tim Leiner

**Affiliations:** Department of Radiology (E01.132), University Medical Center, Heidelberglaan 100, 3584 CX Utrecht, The Netherlands; Institute of Clinical Radiology, Ludwig-Maximilians-University Hospital, Marchioninistrasse 15, 81377 Munich, Germany; Division of Image Processing, Department of Radiology, 1-C2S Leiden University Medical Center, PO Box 9600, 2300 RC Leiden, The Netherlands; Philips Healthcare, Veenpluis 4-6, 5684PC Best, The Netherlands; Julius Center for Health Sciences and Primary Care, University Medical Center Utrecht, Heidelberglaan 100, 3584 CX Utrecht, The Netherlands; Laboratory of Experimental Cardiology, University Medical Center Utrecht, Heidelberglaan 100, 3584 CX Utrecht, The Netherlands; Laboratory of Clinical Chemistry and Hematology, University Medical Center Utrecht, Heidelberglaan 100, 3584 CX Utrecht, The Netherlands

**Keywords:** 3D, Black-blood, CMR, Reproducibility, Young, Healthy, Atherosclerosis, Aorta, Characteristics

## Abstract

**Background:**

Pre-clinical detection of atherosclerosis enables personalized preventive strategies in asymptomatic individuals. Cardiovascular magnetic resonance (CMR) has evolved as an attractive imaging modality for studying atherosclerosis in vivo. Yet, the majority of aortic CMR studies and proposed sequences to date have been performed at 1.5 tesla using 2D BB techniques and a slice thickness of 4–5 mm. Here, we evaluate for the first time the reproducibility of an isotropic, T1-weighted, three-dimensional, black-blood, CMR VISTA sequence (3D-T1-BB-VISTA) for quantification of aortic wall characteristics in healthy, young adults.

**Methods:**

In 20 healthy, young adults (10 males, mean age 31.3 years) of the AMBITYON cohort study the descending thoracic aorta was imaged with a 3.0 T MR system using the 3D-T1-BB-VISTA sequence. The inter-scan, inter-rater and intra-rater reproducibility of aortic lumen, total vessel and wall area and mean and maximum wall thickness was evaluated using Bland-Altman analyses and Intraclass Correlation Coefficients (ICC). Based on these findings, sample sizes for detecting differences in aortic wall characteristics between groups were calculated.

**Results:**

For each studied parameter, the inter-scan, inter-rater and intra-rater reproducibility was excellent as indicated by narrow limits of agreement and high ICCs (ranging from 0.76 to 0.99). Sample sizes required to detect a 5 % difference in aortic wall characteristics between two groups were 203, 126, 136, 68 and 153 per group for lumen area, total vessel area and vessel wall area and for mean and maximum vessel wall thickness, respectively.

**Conclusion:**

The 3D-T1-BB-VISTA sequence provides excellent reproducibility for quantification of aortic wall characteristics and can detect small differences between groups with reasonable sample sizes. Hence, it may be a valuable tool for assessment of the subtle vascular wall changes of early atherosclerosis in asymptomatic populations.

**Electronic supplementary material:**

The online version of this article (doi:10.1186/s12968-016-0237-2) contains supplementary material, which is available to authorized users.

## Background

Cardiovascular disease (CVD) remains a major cause of morbidity and mortality worldwide [1]. Atherosclerosis, the underlying pathophysiological condition, is a slowly progressive, inflammatory process that causes adverse remodelling of the arterial wall. The slowly progressive changes in the arterial wall attributed to atherosclerosis provide a window of opportunity for its pre-clinical detection.

Cardiovascular magnetic resonance (CMR) has evolved as an attractive imaging modality for studying atherosclerosis in vivo [2–4]. The descending and abdominal aorta, the artery where atherosclerosis is known to develop first [5], has been the subject of a number of studies using MR [6–8]. To date, most CMR studies have used 2D sequences at a field strength of 1.5 tesla (T), limiting both spatial resolution and aortic coverage due to time constraints. Nowadays, the widespread availability of 3 T MR scanners has led to increased signal-to-noise ratio vessel wall imaging [9]. Furthermore, there is ongoing development of sequences that aim to optimize image quality whilst covering a larger extent of the aorta with clinically acceptable scan duration [10–12].

One of the most promising CMR sequences that is increasingly being applied in various vascular areas is a 3-dimensional (3D), T1-weighted (T1), black-blood (BB), turbo-spin-echo (TSE) sequence with variable flip angles (3D-T1-BB-VISTA) [6, 13]. This sequence allows for an isotropic depiction of the vessel wall in an acceptable scan time with (sub)-millimetre spatial resolution. Because of the T1-weighting, the VISTA sequence is also sensitive to the effects of contrast agent administration [13]. Hence, the 3D-T1-BB-VISTA sequence may be attractive for the detection of atherosclerosis in early life, when vascular wall abnormalities are still subtle.

The reproducibility of the 3D-T1-BB-VISTA sequence has been evaluated before, yet in an older population with known CVD and using CMR hardware from a different vendor as compared to the current study [6]. The objective of this study was to investigate the inter-scan and inter-rater as well as intra-rater reproducibility of 3D-T1-BB-VISTA CMR in healthy, young adults for assessment of vascular wall characteristics of the descending thoracic aorta. Based on the reproducibility, sample sizes for (therapeutic) trials were calculated by determining the number of participants needed to detect pre-specified differences in aortic wall characteristics between groups.

## Methods

### Study population and design

The study cohort consisted of healthy, young adults participating in the Atherosclerosis Monitoring and Biomarker measurements In The YOuNg (AMBITYON) study. The AMBITYON study, initiated in 2014, is a prospective, ongoing, single centre cohort study that evaluates the interplay between classical cardiovascular risk factors, plasma markers of inflammation, markers of activated circulating cells and atherosclerosis burden (visualized using CMR) in the development of atherosclerosis in young adulthood. The aim of the AMBITYON study is to identify key drivers of atherosclerosis and individuals who are at high risk of developing clinically manifest atherosclerosis later in life. The AMBITYON study is registered in the Netherlands National Trial Register under number 4742. The AMBITYON study currently comprises approximately 130 healthy, young adults who live in a domestic area near the city of Utrecht, Leidsche Rijn. Individuals were eligible for participation in the AMBITYON study if they were between 25 and 35 years of age, did not have a prior history of CVD and did not use cardiovascular preventive medication. In addition, participants with cardiac arrhythmias or with absolute or relative contra-indications to MR were excluded. For the present study, 20 out of 130 AMBITYON participants were randomly selected. The institutional review board of the University Medical Center Utrecht approved the study and written informed consent, including consent to publish, was obtained from all participants before enrolment.

**Fig. 1:**
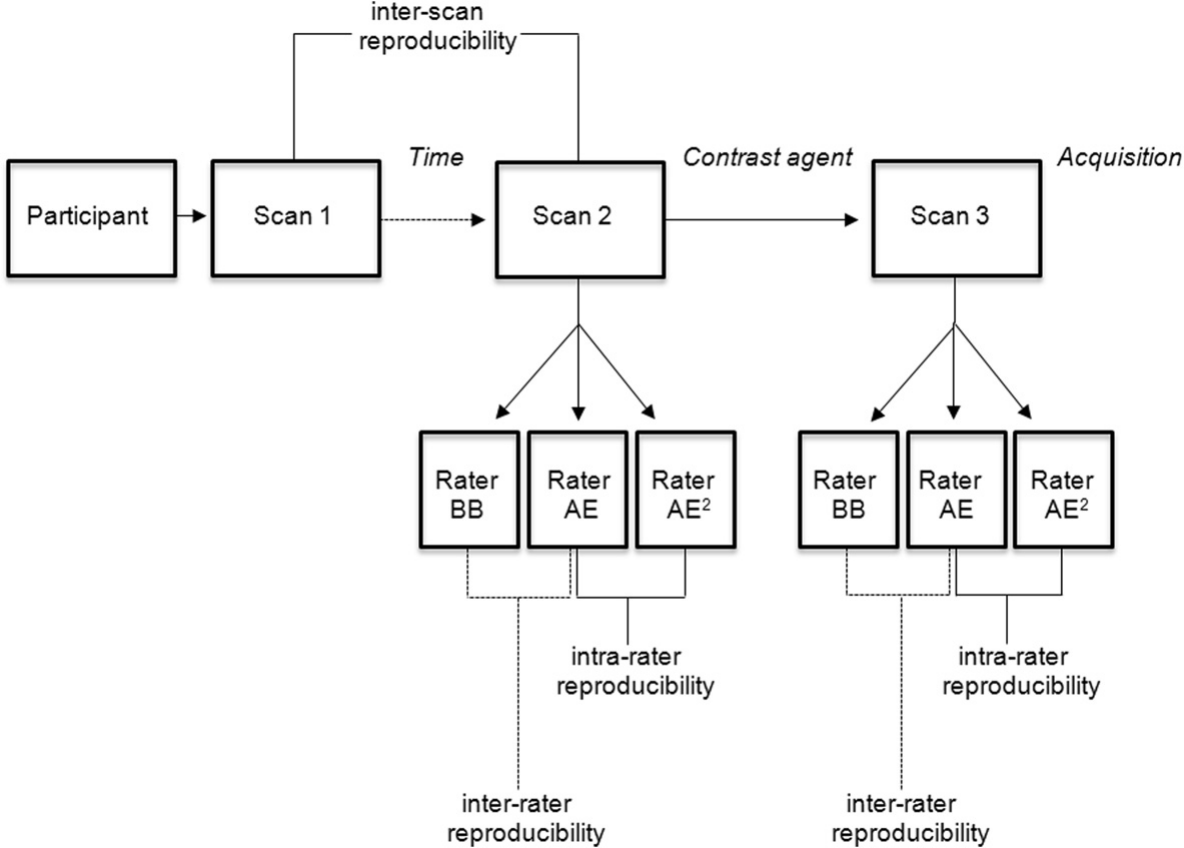
Flow chart of study design. Graphic overview of study design and of inter-scan and non-contrast enhanced and contrast-enhanced inter-rater and intra-rater image analysis to determine reproducibility

The study design is presented in Fig. 1. In short, we acquired three 3D-T1-BB-VISTA scans of the descending thoracic aorta; all scans were performed on the same day. First, a non-contrast enhanced scan was performed (scan 1). Subsequently, participant and table were repositioned and new survey and calibration scans were acquired. Thereafter, a second non-contrast enhanced scan was performed (scan 2). After the second scan, a third late gadolinium enhanced (contrast-enhanced) scan was performed, without repositioning or re-planning, 10 minutes after administration of 0.1 mmol/kg gadobutrol (Gadovist, Bayer Healthcare, Berlin, Germany) (scan 3). Scans 1 and 2 were used to evaluate the inter-scan reproducibility of the 3D-T1-BB-VISTA sequence and scans 2 and 3 to assess inter-rater and intra-rater reproducibility (for both the non-contrast enhanced and contrast-enhanced scans). Total imaging time for each 3D-T1-BB-VISTA scan was 7 min and 38 s. Total imaging time for each participant was approximately 60 min.

### CMR protocol

Both CMR scans were performed on a 3.0 T multi-transmit, clinical MR Scanner (Achieva, Software Release 5.1.7, Philips Healthcare, Best, the Netherlands). Images of the descending thoracic aorta were obtained with the subject placed in a supine position using a 32-channel phased array cardiac coil for signal reception. Images were acquired during free breathing without ECG gating. The coil consisted of two flexible coils that are situated posterior and anterior to the participant. The posterior coil part is embedded in a housing mattress that is integrated on the MR table top.

**Fig. 2:**
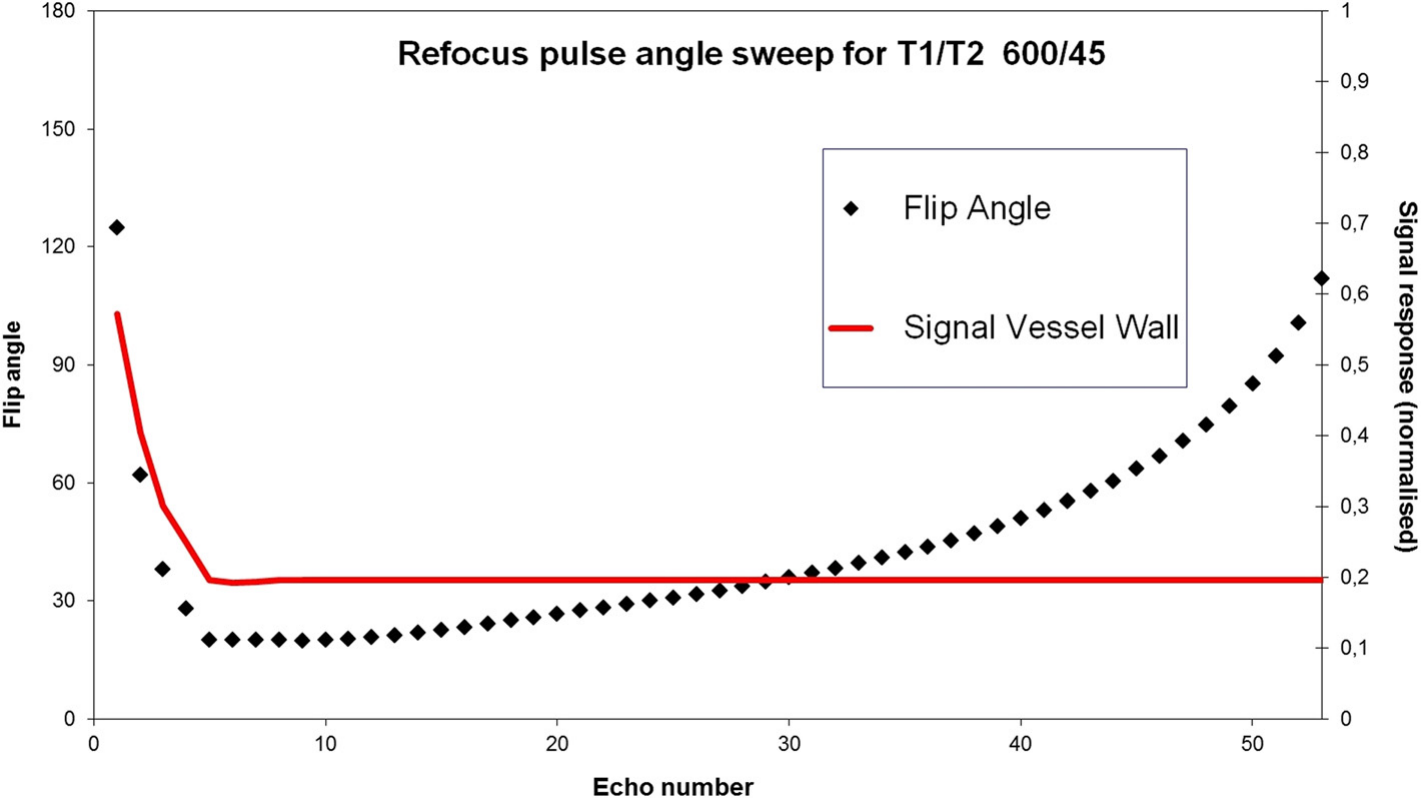
Refocusing flip angle scheme. Refocusing flip angle scheme for 3D-T1-BB-VISTA sequence. Parameters: α_min_: 20°, α_max_: 112°, echo spacing: 3.0 ms, echo train length: 37 + 8 start-up echoes

Following a survey and B1 calibration scan, the descending thoracic aorta was imaged in the sagittal orientation using the 3D-T1-BB-VISTA sequence with Spectral Attenuated Inversion Recovery (SPAIR) fat suppression. All acquisition planes of the survey images were used to locate the descending thoracic aorta and plan the 3D-T1-BB-VISTA sequence. The cranial boundary of the field of view (FOV) was positioned at the top of the aortic arch; the caudal boundary ended at the most distal margin of the cardiac coil, covering approximately 35 cm of aorta. Other imaging parameters of this sequence included: number of slices: 75, FOV: 350x302x45 mm, acquired isotropic voxel size: 1.20×1.20×1.20 mm^3^, reconstructed isotropic voxel size: 0.60×0.60×0.60 mm^3^, matrix: 292×252, echo spacing: 3.0 ms, echo train length: 37 + 8 start-up echoes, refocusing flip angle scheme (Fig. 2; α_min_ = 20°, α_max_ = 112°),TSE factor: 45, TR: 1000 ms, TE: 33 ms, Flip Angle: 90°, number of signal averaging (NSA; used to reduce free induction decay artefacts): 2, slice orientation: sagittal. Additionally, a sensitivity encoding (SENSE) parallel imaging algorithm was used to allow for faster image acquisition. The imaging parameters were held constantly for all three scans. Adequate blood signal suppression was achieved by spins dephasing due to blood flow in the read out direction. To ensure adequate blood suppression under slow flow conditions, 8 start-up echoes were added. This number of start-up echoes was empirically determined in sequence optimization experiments (data not shown) as well as based on a prior study [14].

### CMR image analysis

**Fig. 3:**
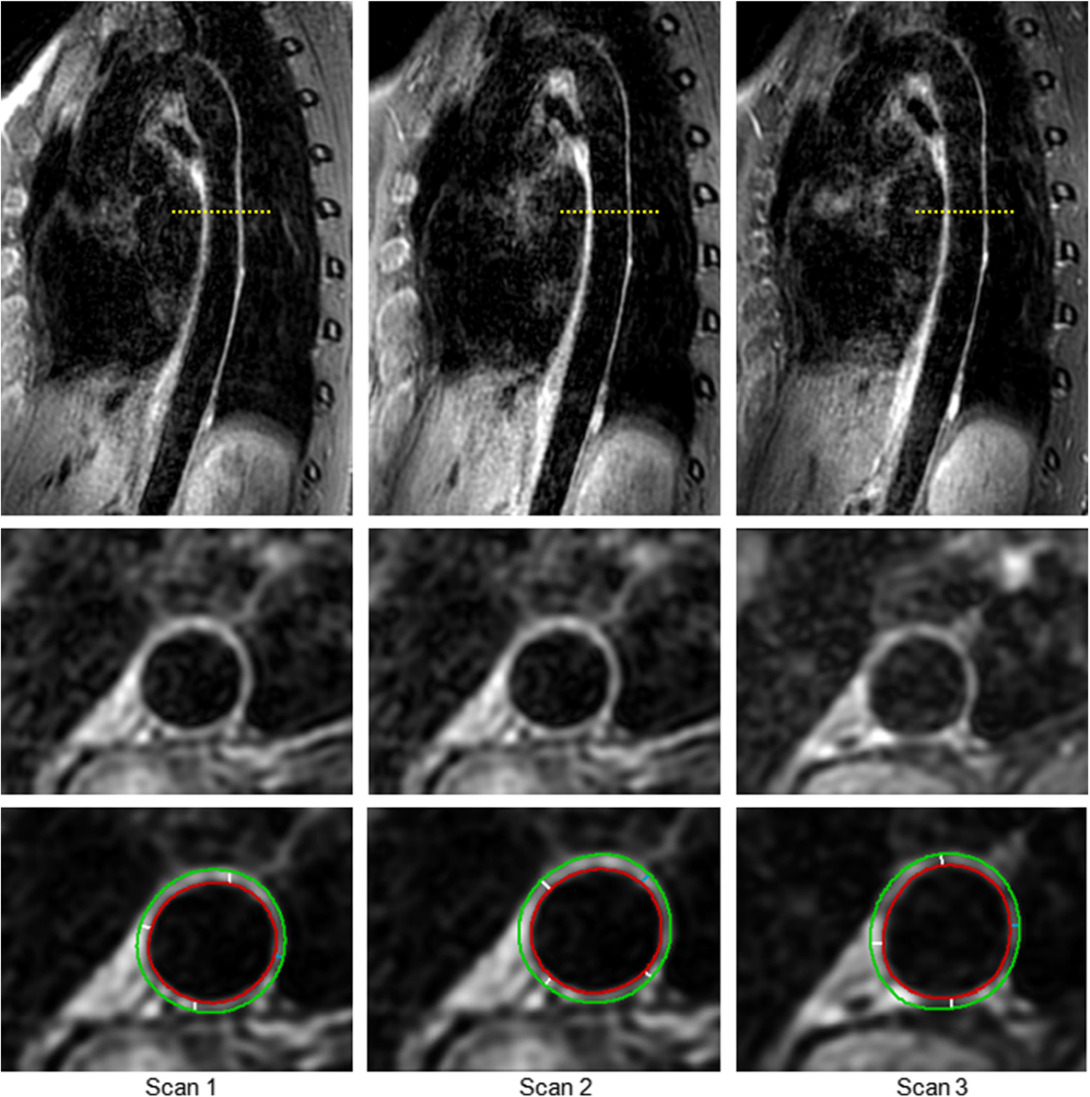
Example of 3D-T1-BB-VISTA acquisition. Examples of the original sagittal and reconstructed plain and traced transverse images of the descending thoracic aorta obtained with the 3D-T1-BB-VISTA sequence in a 32 year old female participant. Acquisitions 1 and scan 2 are identical non-contrast enhanced acquisitions at different time points. Acquisition 3 is a post-contrast acquisition. Aortic wall thicknesses for this slice were 1.52 mm, 1.53 mm and 1.51 mm respectively

The sagittal source images of the 3D-T1-BB-VISTA were reconstructed to approximately 300 slices of 1.2 mm thickness in the transverse plane using customized built-in software (Achieva, Philips Healthcare, Best, the Netherlands). Examples of the original sagittal source images of the 3D-T1-BB-VISTA acquisition as well as reconstructed plain and traced transverse images of all three acquisitions are displayed in Fig. 3. Subsequently, all 3D-T1-BB-VISTA source images and reformations were further analysed using a customized software program designed for measuring geometric dimensions of vascular walls (Vessel Mass, release 5.1, Laboratory for Clinical and Experimental Image processing, The Netherlands) [15]. Due to the limited craniocaudal coverage of the cardiac coil, we analysed the slices from the origin of the descending thoracic aorta to the origin of the celiac trunk. We did not analyse the aortic region below the celiac trunk since the signal to noise ratio (SNR) of this region is insufficient when using a cardiac coil. As such, the image quality is deemed insufficient. In each subject, 1 slice per centimeter of craniocaudal coverage was assessed for geometric parameters. The starting point was the origin of the descending thoracic aorta, just distal to the aortic arch. The endpoint was the last imaging slice before the origin of the first major branch of the abdominal aorta, the celiac trunk. Matching and co-registering of slices across the scans was performed using slice numbers and these two anatomical landmarks.

**Fig. 4:**
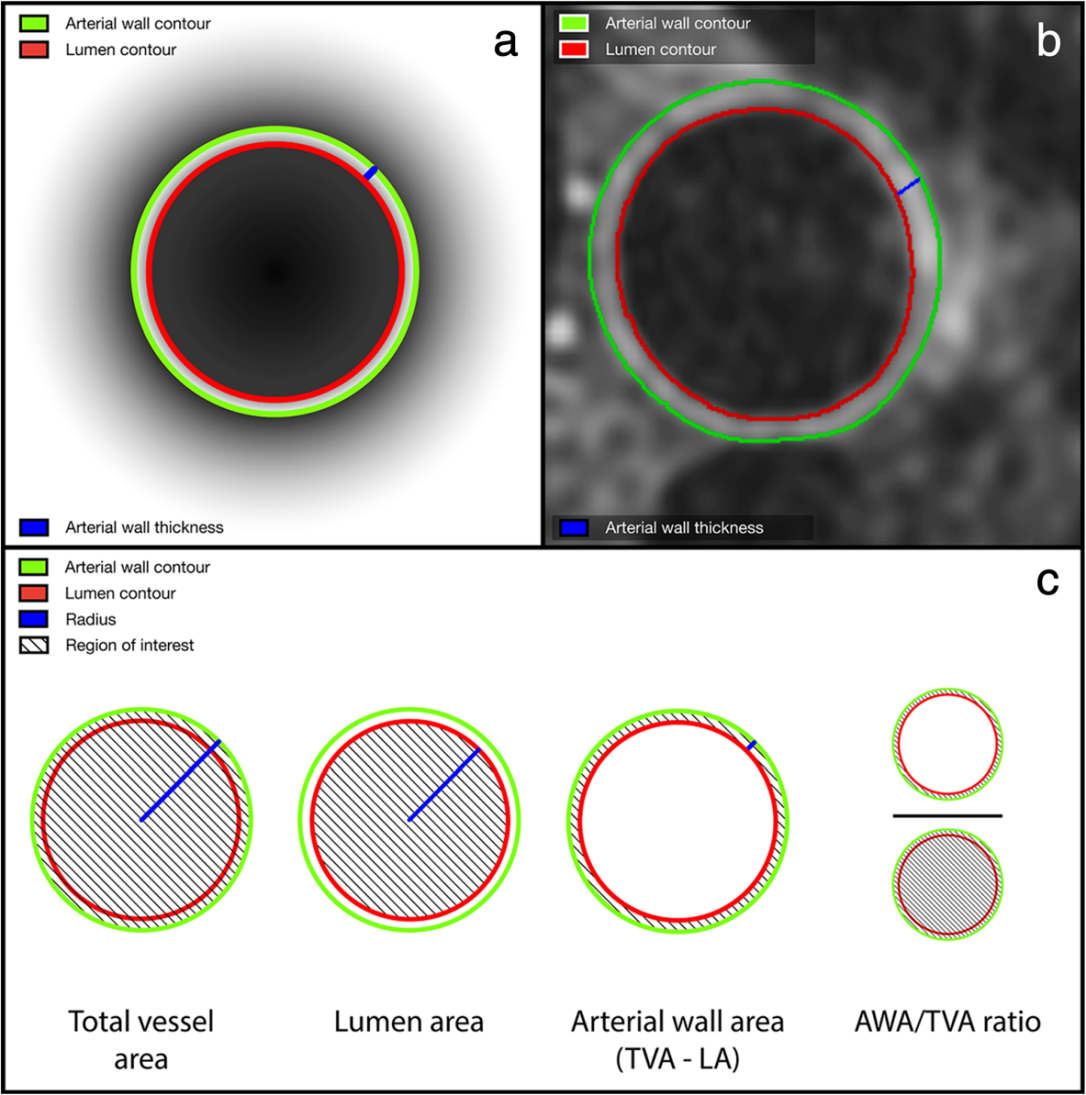
Quantification of aortic wall characteristics. Schematic representation of quantification of descending thoracic aortic wall characteristics. The aortic lumen and outer contours are manually traced. **a** represents a graphic illustration of tracing of the inner and outer contour as well as a thickness measurement. **b** represents an in vivo example of a traced transversal image of the descending thoracic aorta (non-contrast enhanced). **c** represents the definitions and quantification methods of the studied aortic wall characteristics

Images were analysed according to a standardized protocol. First, an experienced radiologist (TL) visually evaluated the selected slices for overall image quality and vascular wall delineation. An image was of sufficient quality if the signal and contrast to noise ratio allowed for a clear visualisation of ≥180° of (i.e., ≥50 %) of the contours of the aortic wall. This method was based on prior studies as well as on the raters’ training set [12, 16]. Insufficient images were removed from analysis. To obtain the most optimal depiction of the aortic wall, we enlarged the remaining images to 300 % of their original proportions and adjusted the brightness and contrast settings (window width: 350–400, image contrast: 40-55 and image brightness: 25–35), all in Vessel Mass. The choice for these features was based on prior studies and the raters’ training set [12, 16–18]. These parameters were held constantly for all analyses. Next, the lumen and outer (adventitial) contours of each image were manually traced. Based on these contours, the software program automatically calculated for each analysed slice the aortic lumen, aortic total vessel and aortic wall areas (cm^2^) and the mean and maximum aortic wall thickness (mm). Vessel Mass measures wall thickness based on the centreline method, which has previously been validated [15]. In short, wall thickness measurements are acquired at 100 equally spaced positions along the aortic wall circumference. This generates the mean aortic wall thickness for each slice. An example is displayed in Fig. 3. For each participant, the mean of each aortic wall parameter was calculated by summing the value for all slices and dividing it by the number of analysed slices. Additionally, to adjust for differences in arterial size within each participant, a normalized wall index, which is the ratio between the aortic wall and the aortic total vessel area (wall area/total vessel area), was calculated. An illustration of aortic wall characteristic quantification is displayed in Fig. 4

.

### Inter-scan and inter-rater and intra-rater reproducibility

To determine inter-scan reproducibility, an experienced rater (AE, 2 years of image analysis experience), analysed the images of scans 1 and 2 in all participants in a computer-based random order. In addition, for determination of non-contrast enhanced intra-rater reproducibility, AE analysed the images of scan 2 for a second time in 10 of the 20 participants. Furthermore, for determination of intra-rater reproducibility of the contrast-enhanced scan (scan 3), AE analysed the images of scan 3 twice in the same 10 subjects. These 10 subjects were chosen using a computer-based random number generator that produced 10 numbers between 1 and 20. Recall bias was reduced to a minimum by using a time frame of approximately 90 days between the first and second image analysis.

To determine inter-rater reproducibility, another rater (BB, 3 years of image analysis experience) independently analysed the images of the 3D-T1-BB-VISTA scans 2 and 3 in the same 10 subjects as AE (Fig. 1). Except for study number, both raters were blinded for participant characteristics and each other’s results. The blinding was assured by replacing each participant’s image identifier with a pre-assigned unique study number. For the inter-scan, inter-rater and intra-rater reproducibility, matching and co-registering of slices across the scans was performed using slice numbers and the two anatomical landmarks.

### Data analysis

#### Baseline

All aortic wall characteristics under study were normally distributed and thus expressed as means ± standard deviation (SD). The aortic wall characteristics are presented at participant level.

#### Reproducibility

Inter-scan, inter-rater and intra-rater reproducibility were evaluated for all aortic wall characteristics under study. First, absolute agreement was assessed using Bland-Altman analysis. In addition, Intra-class Correlation Coefficients (ICC) with 95 % confidence intervals were calculated. The ICC was based on a 2 way random effects model of variance for assessing absolute agreement of single measures. An ICC of <0.4 was considered to indicate a poor reproducibility, an ICC between 0.4 and 0.75 was considered to indicate a fair to good reproducibility and an ICC > 0.75 was considered to indicate an excellent reproducibility. All reproducibility analyses were performed at participant level and carried out using SPSS version 21.0 (IBM, Armonk, IL, USA).

#### Sample size calculations

The means and SDs of the aortic wall characteristics under study were used to calculate the sample size needed to detect a difference of 15 %, 12 %, 10 %, 7 % or 5 % between 2 groups in each of the studied aortic wall characteristics. The choice of these values was based on published literature and the range of expected differences in aortic vessel wall parameters [2, 19]. Each value was corrected for its accompanying inter-scan difference. Sample size calculations were based on a two-sided unpaired *t*-test and performed with a power of 80 % and an alpha level of 5 %. Sample sizes were calculated using PASS version 14.0 (NCSS Statistical Software, Kaysville, UT, USA).

## Results

### Image analysis characteristics

Each participant completed all three 3D-T1-BB-VISTA scans; none of the selected images were excluded for analysis due to poor image quality. On average, the length of the descending thoracic aorta from its origin to celiac trunk was 22 cm. Since 1 slice per centimetre of craniocaudal coverage was analysed, on average 22 slices were analysed per participant. In total, 447 slices were analysed for the inter-scan reproducibility and 203 slices for both the inter-rater and intra-rater reproducibility (for both the non-contrast enhanced and contrast-enhanced acquisitions).

### Participant and aortic wall characteristics

The mean age of the participants was 31.3 years (±2.98); 50 % was male (*n* = 10). The mean values and SDs of the aortic wall characteristics under study are presented in Table 1. Of note, only the mean values of the mean aortic lumen and total vessel area were significantly different between the non-contrast enhanced and contrast-enhanced acquisitions.

**Table 1:** Baseline characteristics of participants (*n =* 20)

	Non-contrast enhanced aortic wall characteristics	Contrast-enhanced aortic wall characteristics
	Mean (SD)^a^	Mean (SD)^a^
- Mean aortic lumen area (cm^2^)^a,b^	2.52 (0.38)	2.63 (0.40)
- Mean aortic total vessel area (cm^2^)^a,b^	3.54 (0.47)	3.66 (0.49)
- Mean aortic wall area (cm^2^)^a^	1.02 (0.12)	1.05 (0.13)
- Mean aortic wall thickness (mm)^a^	1.66 (0.15)	1.67 (0.15)
- Maximum aortic wall thickness (mm)^a^	2.28 (0.23)	2.28 (0.28)
- Mean aortic WA/TVA ratio^a^	0.29 (0.02)	0.29 (0.03)

^a^SD: standard deviation. cm: centimeter. mm: millimeter. WA: wall area. TVA: total vessel area

^b^significantly different between non-contrast enhanced and contrast-enhanced aortic wall characteristics

### Inter-scan reproducibility

The results of the inter-scan reproducibility are presented in Table 2 and in Additional file 1 A-F. In summary, the mean differences of the mean aortic wall area and of the mean and maximum aortic wall thickness were 0.01 cm^2^ (±0.03), 0.01 mm (±0.05) and 0.04 mm (±0.17) respectively. Their lower and upper limit (LOA) values ranged from −0.05 to 0.07 cm^2^, from −0.08 to 0.11 mm and from −0.29 to 0.37 mm respectively. The inter-scan ICCs of all aortic wall characteristics were >0.75, indicating excellent reproducibility.

**Table 2:** Inter-scan reproducibility

	ICC^a^	95 % CI^a^	Mean difference (SD)^a^	95 % LOA^a^
- Mean aortic lumen area (cm^2^)^a^	0.98*	0.95, 0.99	−0.02 (0.08)	−0.17, 0.14
- Mean aortic total vessel area (cm^2^)^a^	0.98*	0.96, 0.99	−0.01 (0.08)	−0.17, 0.16
- Mean aortic wall area (cm^2^)^a^	0.97*	0.92, 0.99	0.01 (0.03)	−0.05, 0.07
- Mean aortic wall thickness (mm)^a^	0.94*	0.87, 0.98	0.01 (0.05)	−0.08, 0.11
- Maximum aortic wall thickness (mm)^a^	0.76*	0.50, 0.90	0.04 (0.17)	−0.29, 0.37
- Mean aortic WA/TVA ratio^a^	0.94*	0.87, 0.98	0.00 (0.01)	−0.01, 0.02

* *P* < .001

^a^ICC: intraclass correlation coefficient (two-way random effects model assessing absolute of single measures). CI: confidence interval. LOA: limits of agreement. WA: wall area. TVA: total vessel area

### Inter-rater reproducibility

Table 3 and Additional file 2 and Additional file 3 A-F display the results of the inter-rater reproducibility. In summary, in the non-contrast enhanced images, the mean difference of the mean aortic wall area was 0.00 cm^2^ (±0.02) (LOA: −0.04, 0.04) as opposed to a mean difference of 0.03 cm^2^ (±0.06) (LOA: −0.10, 0.15) in the contrast-enhanced images. Additionally, the mean differences of the mean and maximum aortic wall thickness in the non-contrast enhanced images were −0.01 mm (±0.02) (LOA: −0.06, 0.03) and 0.14 mm (±0.13) (LOA: −0.11, 0.40) respectively. In the contrast-enhanced images, these values were 0.03 mm (±0.11) (LOA: −0.18, 0.24) and 0.07 mm (±0.12) (LOA: −0.17, 0.31) respectively. All aortic wall characteristics under study were highly reproducible with ICCs of >0.80 for both the non-contrast enhanced as well as the contrast-enhanced acquisitions.

**Table 3:** Inter-rater reproducibility

	ICC^a^	95 % CI^a^	Mean difference (SD)^a^	95 % LOA^a^
Inter-rater (non-contrast enhanced)^a^				
- Mean aortic lumen area (cm^2^)^a^	0.99*	0.88, 1.00	0.05 (0.06)	−0.06, 0.17
- Mean aortic total vessel area (cm^2^)^a^	0.99*	0.93, 1.00	0.05 (0.07)	−0.08, 0.19
- Mean aortic wall area (cm^2^)^a^	0.99*	0.98, 1.00	0.00 (0.02)	−0.04, 0.04
- Mean aortic wall thickness (mm)^a^	0.99*	0.95, 1.00	−0.01 (0.02)	−0.06, 0.03
- Maximum aortic wall thickness (mm)^a^	0.81*	0.40, 0.96	0.14 (0.13)	−0.11, 0.40
- Mean aortic WA/TVA ratio^a^	0.98*	0.76, 0.99	−0.01 (0.00)	−0.02, 0.00
Inter-rater (contrast-enhanced)				
- Mean aortic lumen area (cm^2^)^a^	0.98*	0.92, 1.00	0.04 (0.08)	−0.11, 0.20
- Mean aortic total vessel area (cm^2^)^a^	0.98*	0.91, 1.00	0.06 (0.08)	−0.10, 0.22
- Mean aortic wall area (cm^2^)^a^	0.91*	0.70, 0.98	0.03 (0.06)	−0.10, 0.15
- Mean aortic wall thickness (mm)^a^	0.81*	0.42, 0.95	0.03 (0.11)	−0.18, 0.24
- Maximum aortic wall thickness (mm)^a^	0.88*	0.56, 0.97	0.07 (0.12)	−0.17, 0.31
- Mean aortic WA/TVA ratio^a^	0.81*	0.40, 0.95	0.00 (0.02)	−0.03, 0.04

*P < .001

^a^ICC: intraclass correlation coefficient (two-way random effects model assessing absolute of single measures). CI: confidence interval. LOA: limits of. WA: wall area. TVA: total vessel area

### Intra-rater reproducibility

Table 4 and Additional file 4 and Additional file 5 A-F summarize the Bland-Altman analyses and ICCs for the intra-rater reproducibility. In summary, in the non-contrast enhanced images, the mean difference of the mean aortic wall area was 0.01 cm^2^ (±0.03) (LOA: −0.05, 0.07) as compared to a mean difference of 0.02 cm^2^ (±0.02) (LOA: −0.03, 0.07) in the contrast-enhanced images. Furthermore, in the non-contrast enhanced images, the mean differences of the mean and maximum aortic wall thickness were 0.01 mm (±0.06) (LOA: −0.10, 0.13) and 0.05 mm (±0.11) (LOA: −0.16, 0.25), respectively. In the contrast-enhanced scans these values were 0.03 mm (±0.05) (LOA: −0.07, 0.12) and 0.00 mm (±0.08) (LOA: −0.16, 0.16) respectively. Additionally, the intra-rater ICCs of the studied aortic wall characteristics were all >0.90 for both non-contrast enhanced and contrast-enhanced acquisitions, again indicating excellent reproducibility.

**Table 4:** Intra-rater reproducibility

	ICC^a^	95 % CI^a^	Mean difference (SD)^a^	95 % LOA^a^
Intra-rater (non-contrast enhanced)^a^				
- Mean aortic lumen area (cm^2^)^a^	0.99*	0.95, 1.00	0.02 (0.07)	−0.13, 0.17
- Mean aortic total vessel area (cm^2^)^a^	0.99*	0.96, 1.00	0.04 (0.07)	−0.10, 0.18
- Mean aortic wall area (cm^2^)^a^	0.98*	0.92, 0.99	0.01 (0.03)	−0.05, 0.07
- Mean aortic wall thickness (mm)^a^	0.95*	0.82, 0.99	0.01 (0.06)	−0.10, 0.13
- Maximum aortic wall thickness (mm)^a^	0.92*	0.73, 0.98	0.05 (0.11)	−0.16, 0.25
- Mean aortic WA/TVA ratio^a^	0.91*	0.69, 0.98	0.00 (0.01)	−0.02, 0.02
Intra-rater (contrast-enhanced)^a^				
- Mean aortic lumen area (cm^2^)^a^	0.98*	0.91, 0.99	0.02 (0.09)	−0.17, 0.20
- Mean aortic total vessel area (cm^2^)^a^	0.99*	0.94, 1.00	0.04 (0.09)	−0.13, 0.21
- Mean aortic wall area (cm^2^)^a^	0.98*	0.77, 1.00	0.02 (0.02)	−0.03, 0.07
- Mean aortic wall thickness (mm)^a^	0.93*	0.76, 0.98	0.03 (0.05)	−0.07, 0.12
- Maximum aortic wall thickness (mm)^a^	0.96*	0.83, 0.99	0.00 (0.08)	−0.16, 0.16
- Mean aortic WA/TVA ratio^a^	0.91*	0.71, 0.98	0.00 (0.01)	−0.02, 0.03

* *P* < .001

^a^ICC: intraclass correlation coefficient (two-way random effects model assessing absolute agreement of single measures). CI: confidence interval. LOA: limits of agreement. WA: wall area. TVA: total vessel area

### Sample size calculations

**Fig. 5:**
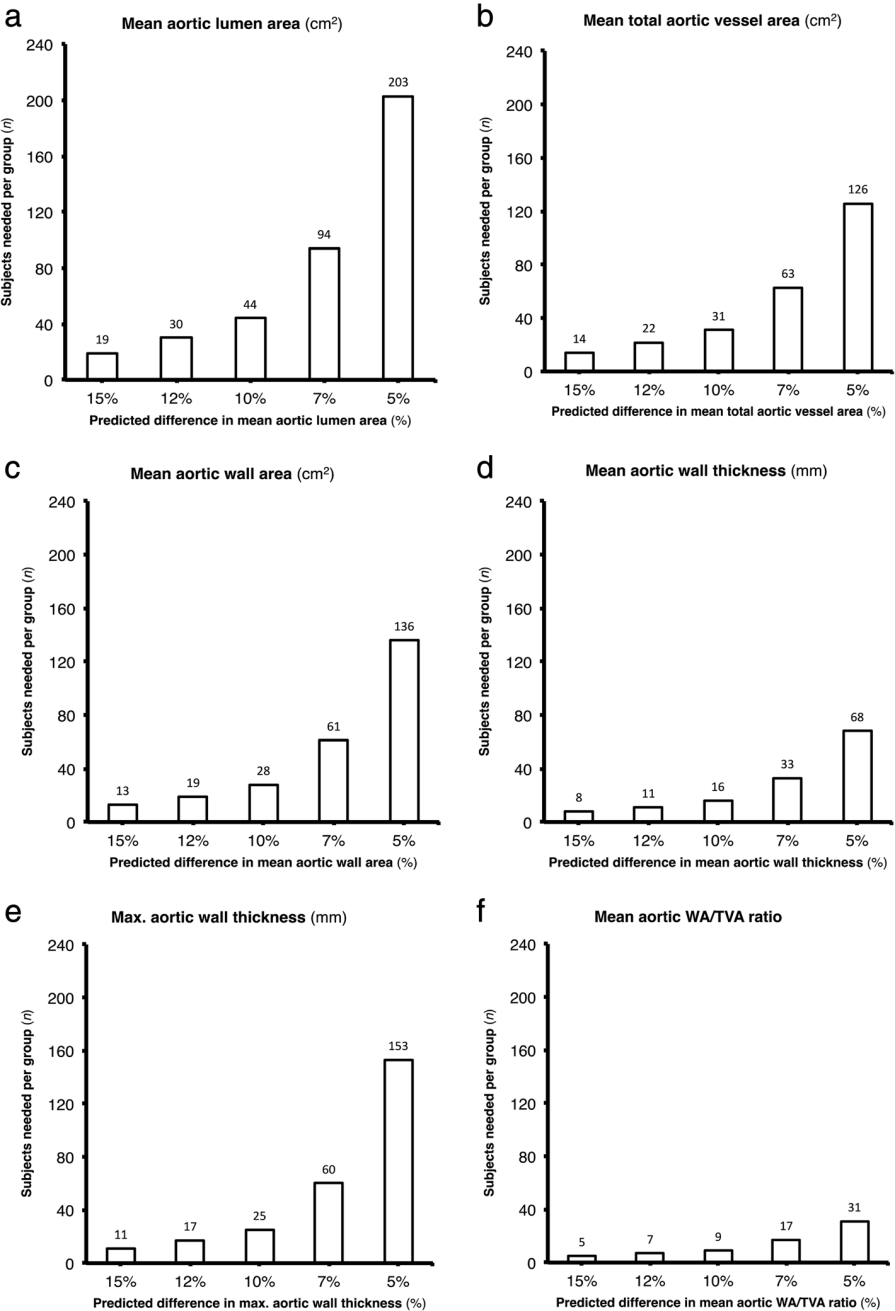
Sample size estimates based on pre-specified differences in aortic wall characteristics between groups. Overview of required sample sizes to detect a mean difference between groups of 15, 12, 10, 7 and 5 % in mean aortic lumen area (**a**), mean total aortic vessel area (**b**), mean aortic wall area (**c**), mean aortic wall thickness (**d**), maximum aortic wall thickness (**e**) and mean aortic WA/TVA ratio (**f**)

In Fig. 5, the results of the sample size calculations are displayed. The sample sizes needed to detect a pre-specified difference of 15 % between groups in mean aortic wall area and mean and maximum aortic wall thickness are 13, 8 and 11 per group respectively. In addition, to detect the smallest pre-specified difference between groups, namely a difference of 5 %, the required sample sizes are 136, 68 and 153 for mean aortic wall area and mean and maximum aortic wall thickness respectively.

## Discussion

The present study demonstrated that the 3D-T1-BB-VISTA sequence is highly reproducible and well-suited for quantification of aortic wall characteristics in young adults. The inter-scan, inter-rater and intra-rater limits of agreement of all aortic wall characteristics under study were narrow and the ICCs were all above 0.75, indicating a low variability in measurement. Sample sizes required to detect a 5 % difference between 2 groups were reasonable for all of the studied aortic wall characteristics. Our results provide an important first step in the process of determining the utility of the 3D-T1-BB-VISTA sequence for preclinical detection and monitoring of atherosclerosis burden in early adult life, when vascular wall abnormalities are still subtle.

The gradually developing lesions in the arterial wall, induced by atherosclerosis, offer an opening for its pre-clinical detection. Unfortunately, currently used CV risk scores are population based, yet do not conclusively identify individuals at high-risk for developing clinically manifest CVD [20]. Furthermore, they are predominantly validated in people over 40 years of age [21]. The American Heart Association (AHA) has recognized this problem and underlined the need for enhanced assessment of non-invasive methods for CVD risk evaluation in young adults [21]. More detailed assessment of the presence or absence of early atherosclerotic changes may improve our understanding of the pathophysiology of atherosclerosis, reveal important determinants and may enhance CVD risk stratification, which is important since identification of high-risk individuals at a young age may enable early interventions to avert or delay the onset of clinically manifest CVD.

Aortic atherosclerosis develops at an early age with the occurrence of aortic wall thickening, a process that precedes the development of discrete plaques by up to several decades. The extent of aortic wall thickening and plaque burden are known to be related to CV risk factors and events [2, 4, 8, 17–19, 22]. Moreover, characteristics of the descending thoracic aortic wall highly relate with those of the carotid arteries [8, 23]. Hence, aortic atherosclerosis is conceived to be a relevant proxy measure of generalized atherosclerosis and possibly of specific interest in young adults.

The assessment of aortic atherosclerosis using imaging modalities is promising [2]. Of all the available modalities for this purpose CMR has various advantages over other imaging techniques. CMR is non-invasive, does not rely on ionizing radiation, and is capable of large anatomical coverage and superior soft tissue contrast that offers the ability to directly visualize and accurately assess various stages of aortic atherosclerosis. Prior studies have demonstrated a good agreement between CMR and histology as well as transesophageal echocardiography with regard to the presence and extent of atherosclerotic plaques [2, 3, 8, 23]. Furthermore, Corti and co-authors suggested, based on their reproducibility data, that 2D CMR is capable of precisely measuring subtle alterations in aortic lesions over time; a change of >5 % in plaque size can be accurately detected [2, 19].

Given the abundance of different CMR sequences, it is desirable to reach consensus on which CMR sequence is most appropriate for early identification and subsequent monitoring of aortic atherosclerosis burden [6–8, 10–12, 22]. Currently, there is an ongoing development of CMR sequences that aim to optimize aortic atherosclerosis quantification. A number of these sequences have shown to be feasible for the quantification of aortic atherosclerosis burden in a highly reproducible manner in healthy subjects and in subjects with CVD [6–8, 10–12, 22]. To date 2D Double Inversion Recovery (DIR) imaging remains the most commonly used CMR technique for evaluation of the aortic wall [11, 24]. The majority of aortic CMR studies and proposed sequences to date have been performed at 1.5 T using 2D BB techniques and a slice thickness of 4-5 mm. Additionally, they provided limited aortic coverage due to time constraints [7, 8, 10–12, 22, 24]. The present study was performed at a 3.0 T system prepared with a dual-source RF-transmission system, allowing for a higher SNR than a 1.5 T system and thus increases spatial resolution [25]. Additionally, this study used a 3D BB sequence. Prior studies have shown that 2D and 3D sequences provide similar morphometric measurements of the aorta. These studies suggest that major benefits of 3D imaging as compared to 2D imaging are the rise in SNR, the ability to reformat the data in any desired orientation with MPR, more detailed coverage of the arterial wall, the reduction in examination duration, the augmented visualization of small plaque constituents and the improved reproducibility (due to smaller slice thicknesses and enlarged flexibility in co-image registration when performing a sequential study) [6, 12, 26–28]. Moreover, a slice thickness of 1.2 mm was used, thereby reducing partial volume effects and providing a high isotropic resolution, which may reduce variability in measurement as compared to sequences that use a larger slice thickness [8]. Hence, in contrast to prior studies, the 3D-T1-BB-VISTA sequence is likely to improve visualization and delineation of the aortic wall and as such allows for an enhanced accuracy and quantification of measurement. In addition, the studied sequence is capable of covering approximately 35 cm of aorta within an acceptable scan time, enabling us to assess its reproducibility, and thus possibly aortic atherosclerosis, over a larger aortic territory and a wide range of aortic sizes. Another advantage of the 3D-T1-BB-VISTA sequence is that it can be used before and after contrast agent administration and thus can directly compare signal intensity before and after contrast agent administration. As such, it may be of value for evaluation of plaque burden, composition and neovascularization [2, 29]. In this study we demonstrated no relevant differences in arterial wall area and thickness between the non-contrast enhanced and contrast-enhanced acquisitions, implying that slow flow artefacts, which possibly decrease the accuracy of measurement, may not be an issue when using the 3D-T1-BB-VISTA sequence in healthy subjects. Yet, this observation does require further evaluation in other cohorts. In addition, a direct comparison of the reproducibility between the 3D-T1-BB-VISTA sequence and 2D sequences in young, healthy individuals remains to be performed.

Mihai and co-workers demonstrated an excellent reproducibility of the 3D-T1-BB-VISTA sequence in 15 patients with CVD and 6 healthy volunteers [6]. Our study extends these preliminary results to a much younger population. As opposed to Mihai and co-workers, the present study did not evaluate the reproducibility of the 3D-T1-BB-VISTA sequence at other locations than the thoracic descending aorta. The reasons for this were that atherosclerosis is known to develop first in the descending aorta and that the SNR of the region below the celiac trunk was insufficient when using a cardiac coil. Yet, since atherosclerosis burden may exhibit different characteristics in other vascular beds, the reproducibility of the 3D-T1-BB-VISTA sequence in the young at other locations than the thoracic descending aorta remains to be determined. Another difference is that the present study was solely performed in healthy young adults, who aortas are not likely to already harbour large atherosclerotic plaques. In contrast, our results confirm the ability of 3D-T1-BB-VISTA to detect subtle differences in aortic wall thickness between subjects with excellent reproducibility. Our findings are in accordance with the results of Mihai and co-workers, who demonstrated an excellent reproducibility of the 3D-T1-BB-VISTA sequence in patients with known CVD [6].

Our study has limitations. First, we did not use ECG triggering or breath holding. Hence, pulsation and breathing artefacts may have introduced slight errors in measurement of absolute aortic wall characteristics. Despite this limitation, reproducibility remained excellent. Nevertheless, tracking of motion using navigators or special motion compensation methods may also improve 3D sequences further. Second, due to time constraints, we analysed one out of every eight slices acquired, thereby inducing a wide gap between two analysed slices. As such, possible focal atherosclerotic lesions may have remained undetected. Yet, since atherosclerosis is conceived to be a generalized disease, we expect the potential loss of information to be minimal in young asymptomatic subjects. Third, our choice of slice thickness created some challenges in preserving adequate signal to allow continuous distinction of aortic wall boundaries in all slices. However, we specifically chose not to use thicker slices since prior studies on 3D BB aorta imaging also used slice thicknesses of ~1 mm with good results [6, 12, 26]. In addition, no slices had to be removed because of insufficient image quality and reproducibility remained excellent between scans as well as between and within raters. Moreover, we believe that the large size of our dataset, our 3D sequence and 3 T system have reduced the potential negative influence of our small slice thickness. Fourth, we did not use a fully automatic image analysis tool for the measurement of the aortic wall characteristics, which made quantification time-consuming and possibly less accurate. Yet, since vascular wall characteristics are usually in the range of a few millimetres, accurate quantification is crucial for its utility in atherosclerosis evaluation. The currently used semi-automatic tool requires dedicated training of raters in image analysis to achieve accurate quantification of aortic wall characteristics, particularly when vascular wall delineation between the vascular wall adventitia and enclosing tissue is unclear. Employment of standardized methods and fully automatic tools may increase efficiency and accuracy of quantification, thereby enabling atherosclerosis detection and monitoring over a larger territory and more homogeneity in measurement across studies. Finally, our results provide an important first step in the process of determining the utility of the 3D-T1-BB-VISTA sequence for preclinical detection and monitoring of atherosclerosis burden in early adult life, when vascular wall abnormalities are still subtle. However, the relation of the studied characteristics with CV risk factors and CVD morbidity and mortality remains to be determined.

## Conclusion

In conclusion 3D-T1-BB-VISTA sequence provides excellent reproducibility for quantification of aortic wall characteristics and can detect differences of 5 % in aortic wall characteristics between groups with a reasonable sample size. It may be a valuable tool for assessment of the subtle vascular wall changes of early atherosclerosis.

## Additional files

Additional file 1: Bland Altman plots for inter-scan reproducibility. (DOC 405 kb) Additional file 2: Bland-Altman plots for non-contrast enhanced inter-rater reproducibility. (DOC 5144 kb) Additional file 3: Bland Altman plots for contrast-enhanced inter-rater reproducibility. (DOC 374 kb) Additional file 4: Bland Altman plots for non-contrast enhanced intra-rater reproducibility. (DOC 388 kb) Additional file 5: Bland Altman plots for contrast-enhanced intra-rater reproducibility. (DOC 378 kb)

## Abbreviations

3D-T1-BB-VISTA: 3 dimensional (3D) black-blood (BB), T1-weighted (T1), turbo-spin-echo sequence with variable flip angles (VISTA)
AMBITYON study: Atherosclerosis Monitoring and Biomarker measurements In The YOuNg
CMR: cardiovascular magnetic resonance
DIR: double inversion recovery
ICC: intra-class correlation coefficient
LOA: lower and upper limit
NSA: number of signal averaging
SD: standard deviation
SENSE: sensitivity encoding
