# Supplementary material for: 3D black blood VISTA vessel wall cardiovascular magnetic resonance of the thoracic aorta wall in young, healthy adults: reproducibility and implications for efficacy trial sample sizes: a cross-sectional study
Source: J Cardiovasc Magn Reson. 2016 Apr 14;18:20. doi: 10.1186/s12968-016-0237-2 (PMC4831203; doi:10.1186/s12968-016-0237-2)
Supplement: Additional file 3: — Bland Altman plots for contrast-enhanced inter-rater reproducibility. (DOC 374 kb) [file 12968_2016_237_MOESM3_ESM.doc]

**ADDITIONAL FILE 3 – Bland Altman plots for contrast-enhanced inter-rater reproducibility**

| A  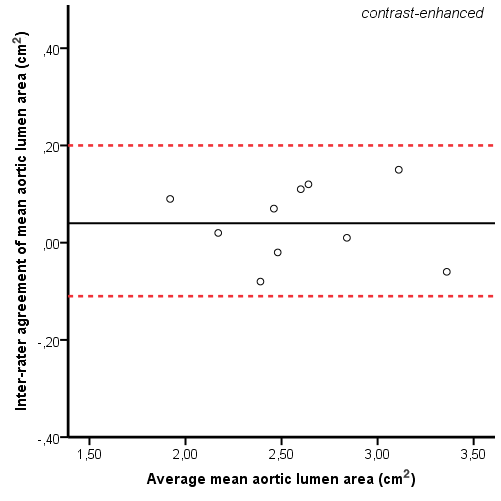 | B  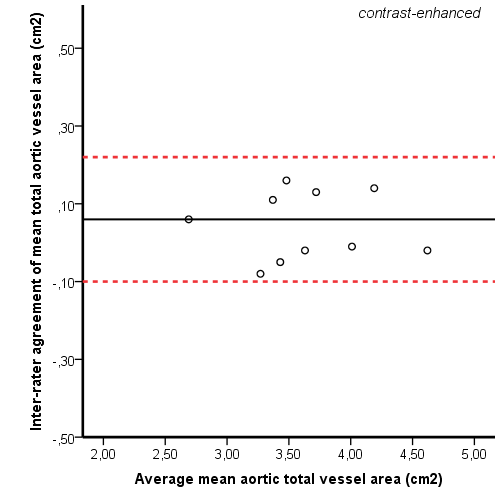 |
| --- | --- |
| C | D |
| E  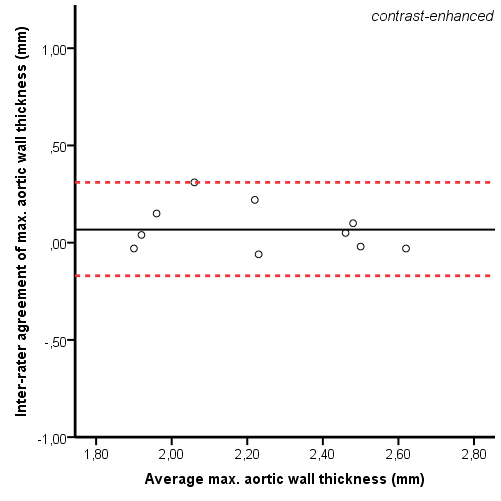 | F  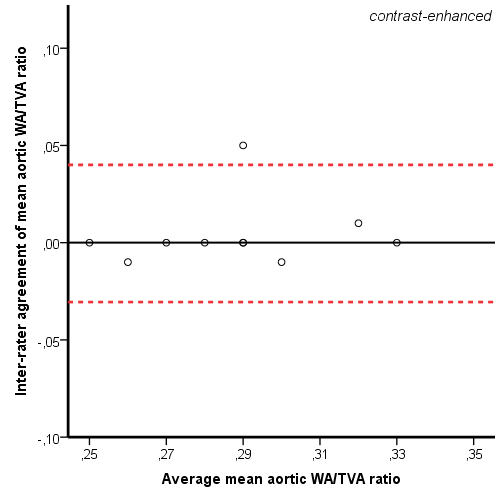 |

Plots of the mean difference between two measurements against the means of these two measurements. The black horizontal line represents the mean difference; the red dotted lines represent the lower- and upper limits (LOA; 1.96xSD of the mean difference).
